# Supplementary material for: Cysteamine–bicalutamide combination therapy corrects proximal tubule phenotype in cystinosis
Source: EMBO Mol Med. 2021 Jun 24;13(7):e13067. doi: 10.15252/emmm.202013067 (PMC8261496; doi:10.15252/emmm.202013067)
Supplement: Supplementary file 1 — Appendix [file EMMM-13-e13067-s002.pdf]

Cysteamine-bicalutamide combination therapy corrects proximal tubule phenotype in cystinosis

Amer Jamalpoor[1], Charlotte AGH van Gelder[2,3‡], Fjodor A Yousef Yengej[4,5‡], Esther A Zaal[2], Sante Princiero Berlingerio[6], Koenraad R Veys[6], Carla Pou Casellas[1], Koen Voskuil[1], Khaled Essa[1], Carola ME Ammerlaan[4,5], Laura Rita Rega[7], Reini van der Welle[8], Marc R Lilien[9], Maarten B Rookmaaker[5], Hans Clevers[4], Judith Klumperman[8], Elena Levtchenko[6], Celia R Berkers[2,10], Marianne C Verhaar[5], Maarten Altelaar[2,3], Rosalinde Masereeuw[1] and Manoe J Janssen[1*]

***Corresponding author**
Manoe J Janssen,
Utrecht University, Faculty of Science, Department of Pharmaceutical Sciences, Division of Pharmacology,
3584 CG, Utrecht, The Netherlands. Email: manoe.janssen@gmail.com

Running title
Combination treatment for cystinosis

APPENDIX

Table of content: **Page**

Appendix Figure S1. Heatmap analysis of metabolites 2
Appendix Figure S2. Identification of robustly changed metabolites in *CTNS* null cells 3
Appendix Table S1. P-values and statistical tests in figures. 4-9

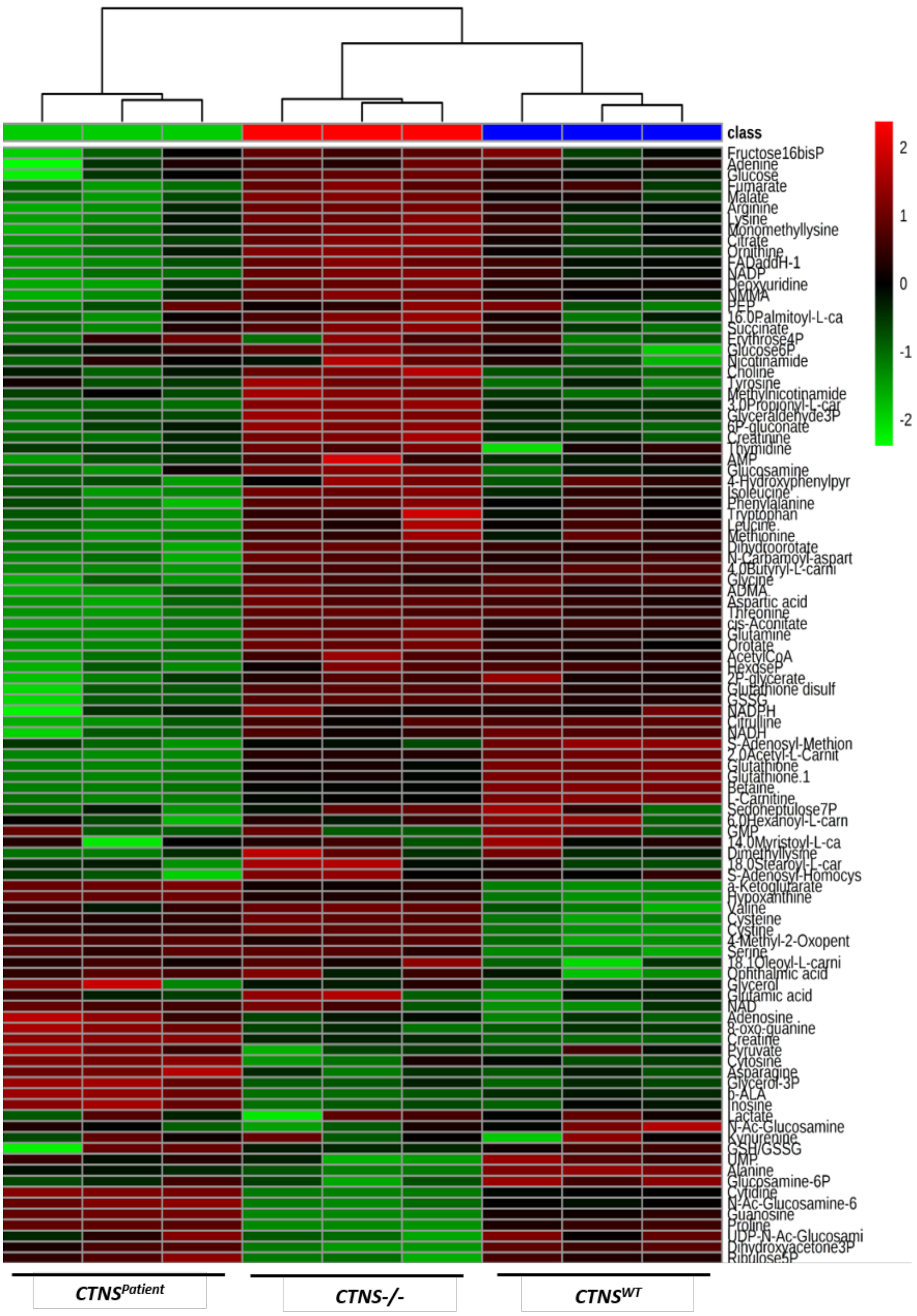

Appendix figure S1. Heatmap analysis of metabolites

Heatmap analysis of metabolites distinctively expressed in control (*CTNS*^{WT}), CRISPR-generated cystinotic cells (*CTNS*$^{-/-}$), and patient-derived cystinotic cells (*CTNS*^{Patient}).

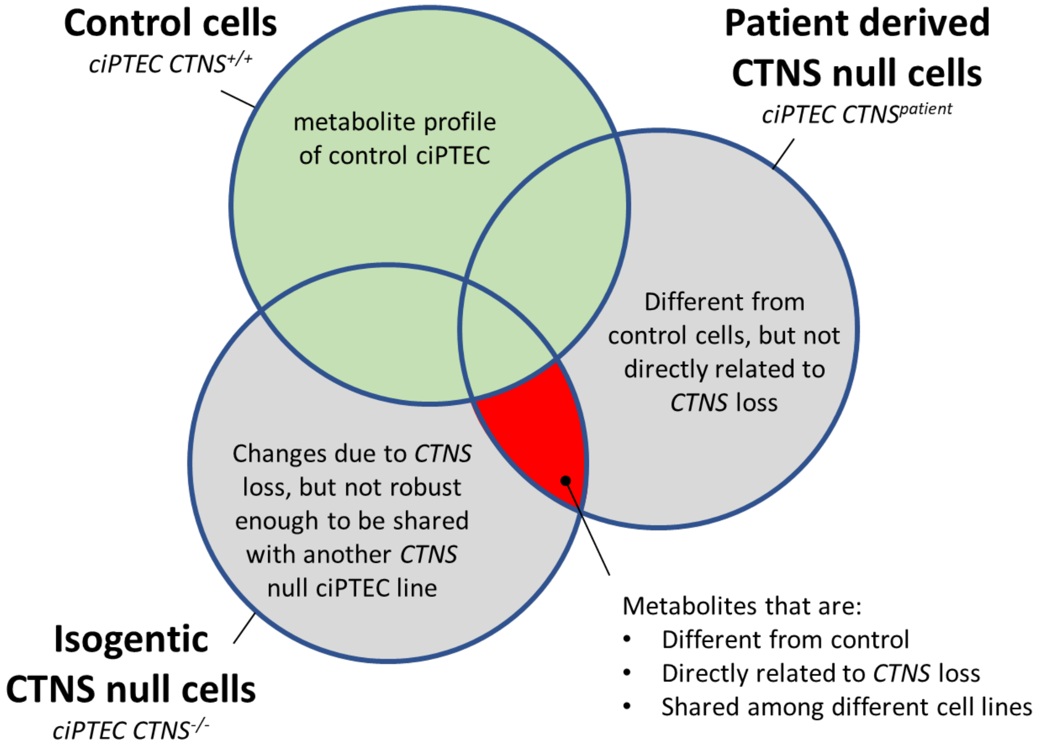

Appendix figure S2. Identification of robustly changed metabolites in *CTNS* null cells

Schematic overview of metabolomic analysis to robustly identify metabolites changed due to CTNS loss.

Appendix Table S1. P-values and statistical tests in figures.

Figure 1	P-Value	comparison	Statistical test
**1A**	<0,0001	CTNSWT VS CTNS-/-	One-way ANOVA
	<0,0001	CTNSWT VS CTNSPatient	One-way ANOVA
**1B**	<0,0001	CTNSWT VS CTNS-/-	One-way ANOVA
	<0,0001	CTNSWT VS CTNSPatient	One-way ANOVA
**1C**	0,0325	CTNSWT VS CTNS-/-	One-way ANOVA
	0,0463	CTNSWT VS CTNSPatient	One-way ANOVA
**1E**	0,1185	Untreated CTNSWT VS untreated CTNSPatient	Unpaired t-test
	0,0028	Bafilomycin treated CTNSWT VS Bafilomycin treated CTNSPatient	Unpaired t-test
**1G**	0,7676	Untreated CTNSWT VS untreated CTNS-/-	Unpaired t-test
	0,3832	Untreated CTNSWT VS untreated CTNSPatient	Unpaired t-test
	0,0174	Bafilomycin treated CTNSWT VS Bafilomycin treated CTNS-/-	Unpaired t-test
	0,0628	Bafilomycin treated CTNSWT VS Bafilomycin treated CTNSPatient	Unpaired t-test
**1H**	0,1496	Untreated CTNSWT VS untreated CTNS-/-	Unpaired t-test
	0,7092	Untreated CTNSWT VS untreated CTNSPatient	Unpaired t-test
	0,0110	Bafilomycin treated CTNSWT VS Bafilomycin treated CTNS-/-	Unpaired t-test
	0,0003	Bafilomycin treated CTNSWT VS Bafilomycin treated CTNSPatient	Unpaired t-test
**1I**	0,0025	CTNSWT VS CTNS-/-	One-way ANOVA
	0,0135	CTNSWT VS CTNSPatient	One-way ANOVA
**1J**	0,5610	CTNSWT VS CTNS-/-	One-way ANOVA
	0,0129	CTNSWT VS CTNSPatient	One-way ANOVA
**Figure 2**	**P-Value**	**comparison**	**Statistical test**
**2D**	<0,0001	CTNSWT VS CTNS-/- - Cystine	Unpaired t-test
	<0,0001	CTNSWT VS CTNSPatient- Cystine	Unpaired t-test
	<0,0001	CTNSWT VS CTNS-/- - Cysteine	Unpaired t-test
	<0,0001	CTNSWT VS CTNSPatient- Cysteine	Unpaired t-test
	<0,0001	CTNSWT VS CTNS-/- - AKG	Unpaired t-test
	<0,0001	CTNSWT VS CTNSPatient- AKG	Unpaired t-test
	<0,0001	CTNSWT VS CTNS-/- - Serine	Unpaired t-test
	<0,0001	CTNSWT VS CTNSPatient- Serine	Unpaired t-test
	<0,0001	CTNSWT VS CTNS-/- - Hypoxanthine	Unpaired t-test
	<0,0001	CTNSWT VS CTNSPatient- Hypoxanthine	Unpaired t-test
	0,0019	CTNSWT VS CTNS-/- - Oleoyl-L-carnitine	Unpaired t-test
	0,0008	CTNSWT VS CTNSPatient- Oleoyl-L-carnitine	Unpaired t-test
	<0,0001	CTNSWT VS CTNS-/- - Betaine	Unpaired t-test
	<0,0001	CTNSWT VS CTNSPatient- Betaine	Unpaired t-test
	0,0298	CTNSWT VS CTNS-/- - L-carnitine	Unpaired t-test
	<0,0001	CTNSWT VS CTNSPatient- L-carnitine	Unpaired t-test
	<0,0001	CTNSWT VS CTNS-/- - Glucosamine-6P	Unpaired t-test
	<0,0001	CTNSWT VS CTNSPatient- Glucosamine-6P	Unpaired t-test
**2E**	0,0095	Control VS Patient	Mann-Whitney test
**2I**	0,0002	CTNSWT VS CTNS-/- - AKGDH	Unpaired t-test
	<0,0001	CTNSWT VS CTNS-/- - LIPA	Unpaired t-test
	0,0210	CTNSWT VS CTNS-/- - ACP2	Unpaired t-test

	0,0118	CTNSWT VS CTNS-/- - CTSS	Unpaired t-test
	0,0023	CTNSWT VS CTNS-/- - CTSC	Unpaired t-test
	0,0001	CTNSWT VS CTNS-/- - IGF2R	Unpaired t-test
	0,0094	CTNSWT VS CTNS-/- - SORT1	Unpaired t-test
	0,0005	CTNSWT VS CTNS-/- - CAT	Unpaired t-test
	0,0052	CTNSWT VS CTNS-/- - CTSA	Unpaired t-test
	0,0035	CTNSWT VS CTNS-/- - LDH	Unpaired t-test
	0,0001	CTNSWT VS CTNS-/- - SOD	Unpaired t-test
**Figure 3**	**P-Value**	**comparison**	**Statistical test**
**3A**	0,0084	CTNSWT VS CTNS-/-	One-way ANOVA
	0,0049	CTNSWT VS CTNSPatient	One-way ANOVA
**3B**	<0,0001	CTNSWT FED Condition VS CTNSWT Starvation Condition	One-way ANOVA
	0,0004	CTNSWT FED Condition VS CTNSWT DMKG Treated Condition	One-way ANOVA
	0,0007	CTNSWT Starvation Condition VS CTNSWT DMKG Treated Condition	Unpaired t-test
**3C**	<0,0001	CTNS-/- FED Condition VS CTNS-/-Starvation Condition	One-way ANOVA
	<0,0001	CTNS-/- FED Condition VS CTNS-/- DMKG Treated Condition	One-way ANOVA
	0,0028	CTNS-/- Starvation Condition VS CTNS-/- DMKG Treated Condition	Unpaired t-test
**3D**	<0,0001	CTNSPatient FED Condition VS CTNSPatient Starvation Condition	One-way ANOVA
	<0,0001	CTNSPatient FED Condition VS CTNSPatient DMKG treated Condition	One-way ANOVA
	0,0012	CTNSPatient Starvation Condition VS CTNSPatient DMKG treated Condition	Unpaired t-test
**3E**	<0,0001	CTNSWT VS CTNS-/- (Starvation)	One-way ANOVA
	<0,0001	CTNSWT VS CTNSPatient (Starvation)	One-way ANOVA
	<0,0001	CTNSWT VS CTNS-/- (Starvation + DMKG)	One-way ANOVA
	<0,0001	CTNSWT VS CTNSPatient (Starvation + DMKG)	One-way ANOVA
**3G**	<0,0001	CTNSWT VS CTNS-/- (Starvation)	One-way ANOVA
	<0,0001	CTNSWT VS CTNSPatient (Starvation)	One-way ANOVA
	<0,0001	CTNSWT VS CTNS-/- (Starvation + DMKG)	One-way ANOVA
	<0,0001	CTNSWT VS CTNSPatient (Starvation + DMKG)	One-way ANOVA
**3I**	0,0001	CTNSWT FED condition VS CTNSWT BafA1 treated	One-way ANOVA
	0,9517	CTNSWT FED condition VS CTNSWT Starved condition	One-way ANOVA
	0,0998	CTNSWT FED condition VS CTNSWT DMKG treated	One-way ANOVA
	0,0014	CTNS-/- FED condition VS CTNS-/- BafA1 treated	One-way ANOVA
	>0,9999	CTNS-/- FED condition VS CTNS-/- Starved condition	One-way ANOVA
	0,0004	CTNS-/- FED condition VS CTNS-/- DMKG treated	One-way ANOVA
	0,0007	CTNSPatient FED condition VS CTNSPatient BafA1 treated	One-way ANOVA
	0,9694	CTNSPatient FED condition VS CTNSPatient Starved condition	One-way ANOVA
	0,0166	CTNSPatient FED condition VS CTNSPatient DMKG treated	One-way ANOVA
**Figure 4**	**P-Value**	**comparison**	**Statistical test**
**4A**			
**Glutathione**	0,8377	CTNSWT no treatment VS CTNS-/- no treatment	One-way ANOVA
	<0,0001	CTNSWT no treatment VS CTNS-/- cysteamine	One-way ANOVA
	0,0507	CTNSWT no treatment VS CTNS-/- Bicalutamide	One-way ANOVA
	<0,0001	CTNSWT no treatment VS. CTNS-/- Combination	One-way ANOVA
	<0,0001	CTNS-/- Bicalutamide VS CTNS-/- Combination	Unpaired t-test
**Cystine**	<0,0001	CTNSWT no treatment VS CTNS-/- no treatment	One-way ANOVA

	0,2649	CTNSWT no treatment VS CTNS-/- cysteamine	One-way ANOVA
	<0,0001	CTNSWT no treatment VS CTNS-/- Bicalutamide	One-way ANOVA
	0,0013	CTNSWT no treatment VS. CTNS-/- Combination	One-way ANOVA
	<0,0001	CTNS-/- Bicalutamide VS CTNS-/- Combination	Unpaired t-test
**Cysteine**	<0,0001	CTNSWT no treatment VS CTNS-/- no treatment	One-way ANOVA
	0,6000	CTNSWT no treatment VS CTNS-/- cysteamine	One-way ANOVA
	<0,0001	CTNSWT no treatment VS CTNS-/- Bicalutamide	One-way ANOVA
	0,0194	CTNSWT no treatment VS. CTNS-/- Combination	One-way ANOVA
	<0,0001	CTNS-/- Bicalutamide VS CTNS-/- Combination	Unpaired t-test
**AKG**	0,0015	CTNSWT no treatment VS CTNS-/- no treatment	One-way ANOVA
	0,0226	CTNSWT no treatment VS CTNS-/- cysteamine	One-way ANOVA
	<0,0001	CTNSWT no treatment VS CTNS-/- Bicalutamide	One-way ANOVA
	<0,0001	CTNSWT no treatment VS. CTNS-/- Combination	One-way ANOVA
	0,0467	CTNS-/- Bicalutamide VS CTNS-/- Combination	Unpaired t-test
**Serine**	<0,0001	CTNSWT no treatment VS CTNS-/- no treatment	One-way ANOVA
	<0,0001	CTNSWT no treatment VS CTNS-/- cysteamine	One-way ANOVA
	0,4286	CTNSWT no treatment VS CTNS-/- Bicalutamide	One-way ANOVA
	0,7028	CTNSWT no treatment VS. CTNS-/- Combination	One-way ANOVA
**Betaine**	<0,0001	CTNSWT no treatment VS CTNS-/- no treatment	One-way ANOVA
	<0,0001	CTNSWT no treatment VS CTNS-/- cysteamine	One-way ANOVA
	0,0191	CTNSWT no treatment VS CTNS-/- Bicalutamide	One-way ANOVA
	0,0076	CTNSWT no treatment VS. CTNS-/- Combination	One-way ANOVA
**Oleoy-L-Carnitine**	<0,0001	CTNSWT no treatment VS CTNS-/- no treatment	One-way ANOVA
	<0,0001	CTNSWT no treatment VS CTNS-/- cysteamine	One-way ANOVA
	0,0011	CTNSWT no treatment VS CTNS-/- Bicalutamide	One-way ANOVA
	0,0004	CTNSWT no treatment VS. CTNS-/- Combination	One-way ANOVA
**Palmitoyl-L-carnitine**	<0,0001	CTNSWT no treatment VS CTNS-/- no treatment	One-way ANOVA
	<0,0001	CTNSWT no treatment VS CTNS-/- cysteamine	One-way ANOVA
	0,0054	CTNSWT no treatment VS CTNS-/- Bicalutamide	One-way ANOVA
	0,0003	CTNSWT no treatment VS. CTNS-/- Combination	One-way ANOVA
**Malate**	<0,0001	CTNSWT no treatment VS CTNS-/- no treatment	One-way ANOVA
	<0,0001	CTNSWT no treatment VS CTNS-/- cysteamine	One-way ANOVA
	<0,0001	CTNSWT no treatment VS CTNS-/- Bicalutamide	One-way ANOVA
	0,1157	CTNSWT no treatment VS. CTNS-/- Combination	One-way ANOVA
	0,0003	CTNS-/- Bicalutamide VS CTNS-/- Combination	Unpaired t-test
**8-oxo-guanine**	<0,0001	CTNSWT no treatment VS CTNS-/- no treatment	One-way ANOVA
	<0,0001	CTNSWT no treatment VS CTNS-/- cysteamine	One-way ANOVA
	0,0011	CTNSWT no treatment VS CTNS-/- Bicalutamide	One-way ANOVA
	0,0004	CTNSWT no treatment VS. CTNS-/- Combination	One-way ANOVA
**pyruvate**	<0,0001	CTNSWT no treatment VS CTNS-/- no treatment	One-way ANOVA
	0,0126	CTNSWT no treatment VS CTNS-/- cysteamine	One-way ANOVA
	<0,0001	CTNSWT no treatment VS CTNS-/- Bicalutamide	One-way ANOVA
	0,0002	CTNSWT no treatment VS. CTNS-/- Combination	One-way ANOVA
	0,0302	CTNS-/- Bicalutamide VS CTNS-/- Combination	Unpaired t-test
**Citrate**	0,0006	CTNSWT no treatment VS CTNS-/- no treatment	One-way ANOVA
	0,0005	CTNSWT no treatment VS CTNS-/- cysteamine	One-way ANOVA
	0,0003	CTNSWT no treatment VS CTNS-/- Bicalutamide	One-way ANOVA
	0,0744	CTNSWT no treatment VS. CTNS-/- Combination	One-way ANOVA
	0,0063	CTNS-/- Bicalutamide VS CTNS-/- Combination	Unpaired t-test

**Glutamine**	<0,0001	CTNSWT no treatment VS CTNS-/- no treatment	One-way ANOVA
	<0,0001	CTNSWT no treatment VS CTNS-/- cysteamine	One-way ANOVA
	0,5643	CTNSWT no treatment VS CTNS-/- Bicalutamide	One-way ANOVA
	<0,0001	CTNSWT no treatment VS. CTNS-/- Combination	One-way ANOVA
	<0,0001	CTNS-/- Bicalutamide VS CTNS-/- Combination	Unpaired t-test
**Guanine**	0,0097	CTNSWT no treatment VS CTNS-/- no treatment	One-way ANOVA
	0,0021	CTNSWT no treatment VS CTNS-/- cysteamine	One-way ANOVA
	0,9620	CTNSWT no treatment VS CTNS-/- Bicalutamide	One-way ANOVA
	0,8590	CTNSWT no treatment VS. CTNS-/- Combination	One-way ANOVA
**cAMP**	0,0055	CTNSWT no treatment VS CTNS-/- no treatment	One-way ANOVA
	0,0010	CTNSWT no treatment VS CTNS-/- cysteamine	One-way ANOVA
	0,9999	CTNSWT no treatment VS CTNS-/- Bicalutamide	One-way ANOVA
	0,0783	CTNSWT no treatment VS. CTNS-/- Combination	One-way ANOVA
	0,0144	CTNS-/- Bicalutamide VS CTNS-/- Combination	Unpaired t-test
**4E**			
**AKGDH**	0,0331	CTNSWT no treatment VS CTNS-/- no treatment	One-way ANOVA
	0,0030	CTNSWT no treatment VS CTNS-/- cysteamine	One-way ANOVA
	0,0256	CTNSWT no treatment VS CTNS-/- Bicalutamide	One-way ANOVA
	0,0008	CTNSWT no treatment VS. CTNS-/- Combination	One-way ANOVA
**GLUD1;GLUD2**	0,0053	CTNSWT no treatment VS CTNS-/- no treatment	One-way ANOVA
	0,0539	CTNSWT no treatment VS CTNS-/- cysteamine	One-way ANOVA
	0,0197	CTNSWT no treatment VS CTNS-/- Bicalutamide	One-way ANOVA
	0,0810	CTNSWT no treatment VS. CTNS-/- Combination	One-way ANOVA
**IGF2R**	0,0015	CTNSWT no treatment VS CTNS-/- no treatment	One-way ANOVA
	0,0885	CTNSWT no treatment VS CTNS-/- cysteamine	One-way ANOVA
	0,4080	CTNSWT no treatment VS CTNS-/- Bicalutamide	One-way ANOVA
	0,5544	CTNSWT no treatment VS. CTNS-/- Combination	One-way ANOVA
**GSTK1**	0,0008	CTNSWT no treatment VS CTNS-/- no treatment	One-way ANOVA
	0,3489	CTNSWT no treatment VS CTNS-/- cysteamine	One-way ANOVA
	0,9846	CTNSWT no treatment VS CTNS-/- Bicalutamide	One-way ANOVA
	0,7868	CTNSWT no treatment VS. CTNS-/- Combination	One-way ANOVA
**COX6B1**	0,0135	CTNSWT no treatment VS CTNS-/- no treatment	One-way ANOVA
	0,9098	CTNSWT no treatment VS CTNS-/- cysteamine	One-way ANOVA
	0,0864	CTNSWT no treatment VS CTNS-/- Bicalutamide	One-way ANOVA
	0,0057	CTNSWT no treatment VS. CTNS-/- Combination	One-way ANOVA
**ACACA**	0,0005	CTNSWT no treatment VS CTNS-/- no treatment	One-way ANOVA
	<0,0001	CTNSWT no treatment VS CTNS-/- cysteamine	One-way ANOVA
	0,0709	CTNSWT no treatment VS CTNS-/- Bicalutamide	One-way ANOVA
	0,0228	CTNSWT no treatment VS. CTNS-/- Combination	One-way ANOVA
**GLS**	<0,0001	CTNSWT no treatment VS CTNS-/- no treatment	One-way ANOVA
	<0,0001	CTNSWT no treatment VS CTNS-/- cysteamine	One-way ANOVA
	0,0003	CTNSWT no treatment VS CTNS-/- Bicalutamide	One-way ANOVA
	<0,0001	CTNSWT no treatment VS. CTNS-/- Combination	One-way ANOVA
**CASP3**	<0,0001	CTNSWT no treatment VS CTNS-/- no treatment	One-way ANOVA
	<0,0001	CTNSWT no treatment VS CTNS-/- cysteamine	One-way ANOVA
	<0,0001	CTNSWT no treatment VS CTNS-/- Bicalutamide	One-way ANOVA
	<0,0001	CTNSWT no treatment VS. CTNS-/- Combination	One-way ANOVA
**NPC1**	0,0132	CTNSWT no treatment VS CTNS-/- no treatment	One-way ANOVA
	0,0500	CTNSWT no treatment VS CTNS-/- cysteamine	One-way ANOVA
	>0,9999	CTNSWT no treatment VS CTNS-/- Bicalutamide	One-way ANOVA
	0,0490	CTNSWT no treatment VS. CTNS-/- Combination	One-way ANOVA

Figure 5	P-Value	comparison	Statistical test
**5A**	0,0891	CTNS-/- DMKG VS CTNS-/- DMKG and Cysteamine	One-way ANOVA
	0,0002	CTNS-/- DMKG VS CTNS-/- DMKG and Bicalutamide	One-way ANOVA
	0,0013	CTNS-/- DMKG VS CTNS-/- DMKG and Cysteamine and Bicalutamide	One-way ANOVA
**5B**	0,0002	CTNS-/- fed VS CTNS-/- BafA1	One-way ANOVA
	<0,0001	CTNS-/- fed VS CTNS-/- DMKG	One-way ANOVA
	0,0326	CTNS-/- fed VS CTNS-/- DMKG and Bicalutamide	One-way ANOVA
	<0,0001	CTNS-/- fed VS CTNS-/- DMKG and Cysteamine	One-way ANOVA
	0,5640	CTNS-/- fed VS CTNS-/- DMKG and Cysteamine and Bicalutamide	One-way ANOVA
	0,0005	CTNS-/- DMKG VS CTNS-/- DMKG and Bicalutamide	Unpaired t-test
	0,2782	CTNS-/- DMKG VS CTNS-/- DMKG and Cysteamine	Unpaired t-test
	<0,0001	CTNS-/- DMKG VS CTNS-/- DMKG and Cysteamine and Bicalutamide	Unpaired t-test
	0,0497	CTNS-/- DMKG and Bicalutamide VS CTNS-/- DMKG and Cysteamine and Bicalutamide	Unpaired t-test
**5C**	<0,0001	CTNS-/- fed VS CTNS-/- starvation	One-way ANOVA
	<0,0001	CTNS-/- fed VS CTNS-/- DMKG	One-way ANOVA
	<0,0001	CTNS-/- fed VS CTNS-/- DMKG and Bicalutamide	One-way ANOVA
	<0,0001	CTNS-/- fed VS CTNS-/- DMKG and Cysteamine	One-way ANOVA
	<0,0001	CTNS-/- fed VS CTNS-/- DMKG and Cysteamine and Bicalutamide	One-way ANOVA
	<0,0001	CTNS-/- starvation VS CTNS-/- DMKG	Unpaired t-test
	<0,0001	CTNS-/- DMKG VS CTNS-/- DMKG and Bicalutamide	Unpaired t-test
	<0,0001	CTNS-/- DMKG VS CTNS-/- DMKG and Cysteamine	Unpaired t-test
	<0,0001	CTNS-/- DMKG VS CTNS-/- DMKG and Cysteamine and Bicalutamide	Unpaired t-test
	0,8017	CTNS-/- DMKG and Bicalutamide VS CTNS-/- DMKG and Cysteamine	Unpaired t-test
	0,0021	CTNS-/- DMKG and Bicalutamide VS CTNS-/- DMKG and Cysteamine and Bicalutamide	Unpaired t-test
	<0,0001	CTNS-/- DMKG and Cysteamine VS CTNS-/- DMKG and Cysteamine and Bicalutamide	Unpaired t-test
**5E**	0,0007	CTNSWT no Bicalutamide VS CTNSWT with Bicalutamide	One-way ANOVA
	0,0055	CTNSWT no Bicalutamide VS CTNS-/- no Bicalutamide	One-way ANOVA
	0,4194	CTNSWT no Bicalutamide VS CTNS-/- with Bicalutamide	One-way ANOVA
	0,0009	CTNS-/- no Bicalutamide VS CTNS-/- with Bicalutamide	One-way ANOVA
**5F**	<0,0001	CTNSWT VS CTNS-/- NT	One-way ANOVA
	<0,0001	CTNSWT VS CTNS-/- Bicalutamide	One-way ANOVA
	0,0107	CTNSWT VS CTNS-/- Cysteamine	One-way ANOVA
	0,6945	CTNSWT VS CTNS-/- Combination	One-way ANOVA
	0,0304	CTNS-/- Cysteamin VS CTNS-/- Combination	Unpaired t-test
**5G**	<0,0001	CTNSWT VS CTNSPatient NT	One-way ANOVA
	<0,0001	CTNSWT VS CTNSPatient Bicalutamide	One-way ANOVA
	0,0097	CTNSWT VS CTNSPatient Cysteamine	One-way ANOVA
	0,9750	CTNSWT VS CTNSPatient Combination	One-way ANOVA
	<0,0001	CTNSPatient Cysteamine VS CTNSPatient Combination	Unpaired t-test
**Figure 6**	**P-Value**	**comparison**	**Statistical test**
**6B**	0,9987	CTNSWT-1 VS CTNSWT-2	One-way ANOVA

	0,0035	CTNSWT-1 VS CTNSPatient-1	One-way ANOVA
	0,0004	CTNSWT-1 VS CTNSPatient-2	One-way ANOVA
	0,0027	CTNSWT-2 VS CTNSPatient-1	One-way ANOVA
	0,0003	CTNSWT-2 VS CTNSPatient-2	One-way ANOVA
	0,8220	CTNSPatient-1 VS CTNSPatient-2	One-way ANOVA
**6C**	<0,0001	CTNSPatient-1 NT VS CTNSPatient-1 Bicalutamide	One-way ANOVA
	<0,0001	CTNSPatient-1 NT VS CTNSPatient-1 Cysteamine	One-way ANOVA
	<0,0001	CTNSPatient-1 NT VS CTNSPatient-1 Combination	One-way ANOVA
	0,0218	CTNSPatient-1 Cysteamine VS CTNSPatient-1 Combination	Unpaired t-test
**6D**	0,1173	CTNSPatient-2 NT VS CTNSPatient-2 Bicalutamide	One-way ANOVA
	<0,0001	CTNSPatient-2 NT VS CTNSPatient-2 Cysteamine	One-way ANOVA
	<0,0001	CTNSPatient-2 NT VS CTNSPatient-2 Combination	One-way ANOVA
	0,0030	CTNSPatient-2 Cysteamine VS CTNSPatient-2 Combination	Unpaired t-test
**6E**	0,3417	CTNSPatient-1 NT VS CTNSPatient-1 Bicalutamide - AKG level	One-way ANOVA
	0,0671	CTNSPatient-1 NT VS CTNSPatient-1 Cysteamine - AKG level	One-way ANOVA
	0,1765	CTNSPatient-1 NT VS CTNSPatient-1 Combination - AKG level	One-way ANOVA
	0,0033	CTNSPatient-2 NT VS CTNSPatient-2 Bicalutamide - AKG level	One-way ANOVA
	0,5527	CTNSPatient-2 NT VS CTNSPatient-2 Cysteamine - AKG level	One-way ANOVA
	0,0005	CTNSPatient-2 NT VS CTNSPatient-2 Combination - AKG level	One-way ANOVA
**6G**	0,0040	ctnsWT VS ctns-/-	Unpaired t-test
**6H**	0,1088	ctns-/- control VS CTNS-/- Bicalutamide	One-way ANOVA
	0,0153	ctns-/- control VS CTNS-/- Cysteamine	One-way ANOVA
	0,0066	ctns-/- control VS CTNS-/- Combination	One-way ANOVA
	0,0888	CTNS-/- Cysteamine VS CTNS-/- Combination	Unpaired t-test
**Figure EV1**	**P-Value**	**comparison**	**Statistical test**
**EV1C**	<0,0001	CTNSWT VS colony 3	One-way ANOVA
	<0,0001	CTNSWT VS colony 7	One-way ANOVA
	0,0002	CTNSWT VS colony 35	One-way ANOVA
	<0,0001	CTNSWT VS CTNSPatient	One-way ANOVA
**EV1D**	<0,0001	Colony 3 VS colony 3 + cysteamine	Unpaired t-test
	<0,0001	Colony 7 VS colony 3 + cysteamine	Unpaired t-test
	<0,0001	Colony 35 VS colony 3 + cysteamine	Unpaired t-test
	<0,0001	CTNSPatient VS CTNSPatient + cysteamine	Unpaired t-test
**Figure EV4**	**P-Value**	**comparison**	**Statistical test**
**EV4E**	0,0060	CTNSWT no bicalutamide VS CTNSWT with bicalutamide	Unpaired t-test
	0.0581	CTNSWT no bicalutamide VS CTNS-/- no bicalutamide	Unpaired t-test
	0,6237	CTNS-/- no bicalutamide VS CTNS-/- with bicalutamide	Unpaired t-test
**EV4D**	0,0002	CTNS-/- control condition VS CTNS-/- bafilomycin treated	One-way ANOVA
	>0,9999	CTNS-/- control condition VS CTNS-/- starvation	One-way ANOVA
	<0,0001	CTNS-/- control condition VS CTNS-/- DMKG treated	One-way ANOVA
	0,9997	CTNS-/- control condition VS CTNS-/- cysteamine treated	One-way ANOVA
	0,9996	CTNS-/- control condition VS CTNS-/- bicalutamide treated	One-way ANOVA
	0,9997	CTNS-/- control condition VS CTNS-/- bicalutamide and cysteamine treated	One-way ANOVA
**EV4F**	<0,0001	CTNSWT fed VS CTNSWT starvation	One-way ANOVA
	0,0009	CTNSWT fed VS CTNSWT with bicalutamide	One-way ANOVA
