# Supplementary material for: Cysteamine–bicalutamide combination therapy corrects proximal tubule phenotype in cystinosis
Source: EMBO Mol Med. 2021 Jun 24;13(7):e13067. doi: 10.15252/emmm.202013067 (PMC8261496; doi:10.15252/emmm.202013067)
Supplement: Supplementary file 2 — Expanded View Figures PDF [file EMMM-13-e13067-s004.pdf]

Expanded View Figures

Figure EV1. Generation of *CTNS*$^{-/-}$ isogenic cell line of ciPTEC using CRISPR.

A Schematic overview of the CRISPR-based strategy to knockout the *CTNS* gene in ciPTEC.
B Sanger sequencing chromatogram shows resulting sequence in CRISPR-generated cystinotic cells (*CTNS*$^{-/-}$).
C Quantification of cystine levels (nmol/mg protein) by HPLC-MS/MS in control (*CTNS*^{WT}), CRISPR-generated cystinotic cells (*CTNS*$^{-/-}$; line 3, 7 and 35, and patient-derived cystinotic cells (*CTNS*^{Patient}) (n = 3–6).
D Quantification of cystine levels (nmol/mg protein) by HPLC-MS/MS in *CTNS*$^{-/-}$ lines (3, 7 and 35), and *CTNS*^{Patient} cells upon treatment with cysteamine (100 μM) (n = 3–6).

Data information: Data are expressed as mean ± SEM. *P-values < 0.05 were considered to be significant. One-way ANOVA with Dunnett's correction (C and D) or unpaired t-test (D). Exact P-values and statistical tests are listed in Appendix Table S1.

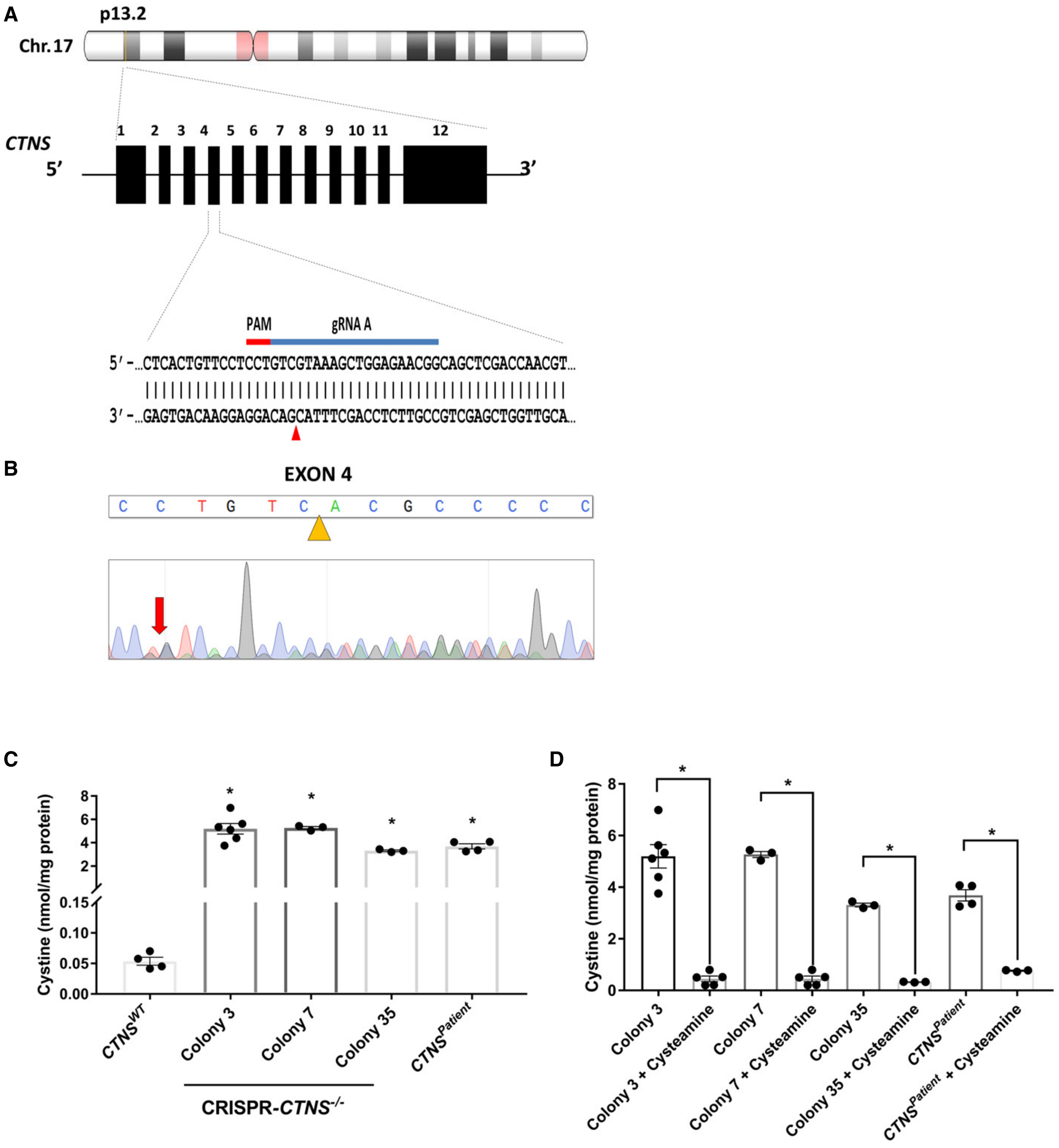

Figure EV1.

Figure EV2. Decreased retention of mTOR on the lysosomal membrane of cystinotic cells.

Representative immunofluorescent staining of *CTNS*^{WT}, *CTNS*$^{-/-}$ and *CTNS*^{Patient} co-immunolabelled with lysosomal-associated membrane protein 1 (LAMP1; green) and mTOR (Red) (n = 3). Merge images with zoomed areas are representative of the localization of mTOR with lysosomes in various experimental conditions. Scale bars are 10 µm.

© 2021 The Authors

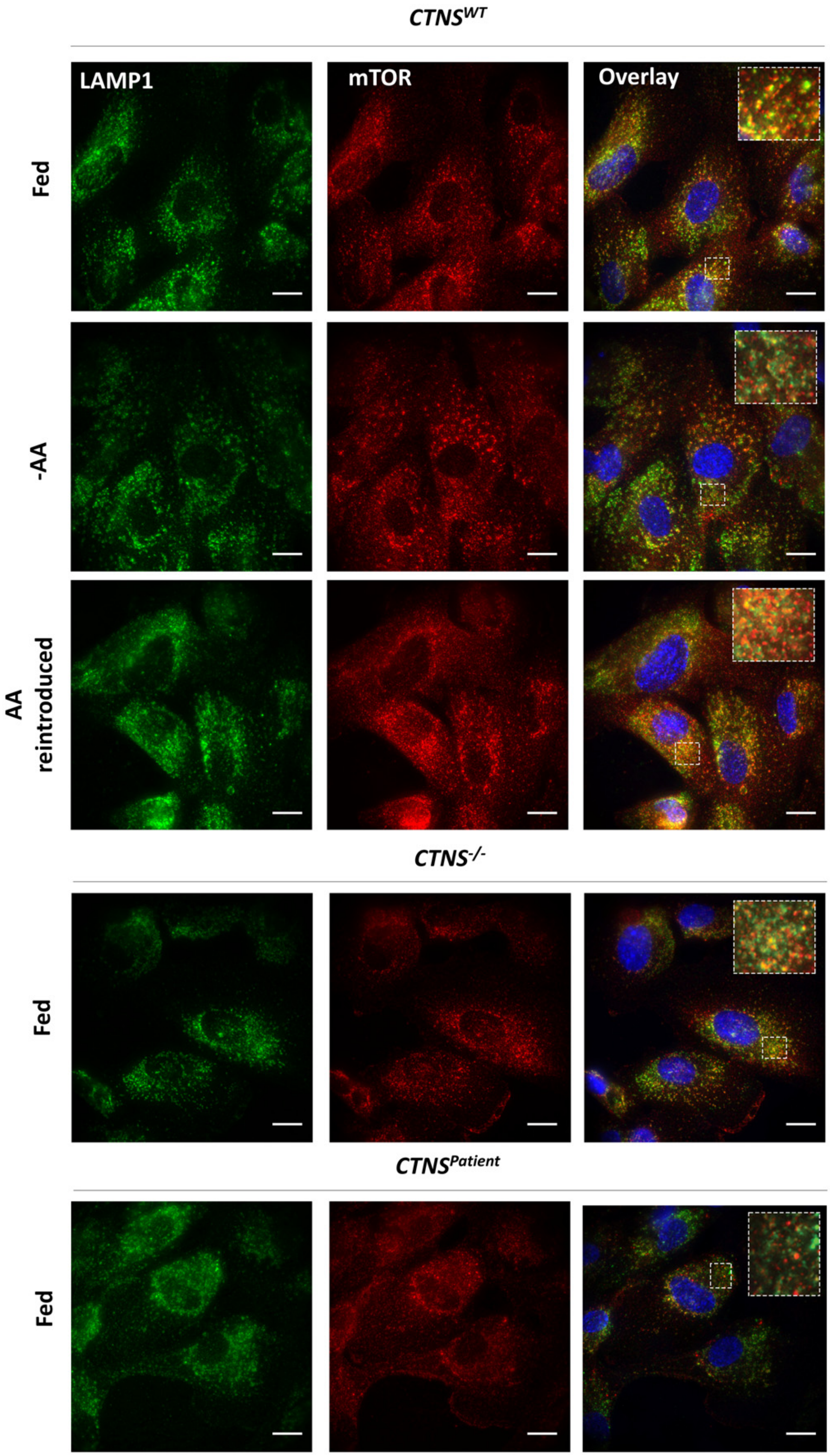
CTNS^WT
LAMP1
mTOR
Overlay
Fed
-AA
AA reintroduced
CTNS-/-
Fed
CTNS^Patient
Fed

Figure EV2.

Figure EV3. Toxicity profile of dimethyl α-ketoglutarate (DMKG) and the drugs tested for the treatment of cystinosis.

A Cell viability curves of dimethyl α-ketoglutarate (DMKG) in *CTNS*^{WT} and *CTNS*$^{-/-}$ cells after 4 h of incubation in fed and starved condition (n = 3).

B, C Cell viability curves of the increasing concentration of DMKG in control (*CTNS*^{WT}) and CRISPR-generated cystinotic (*CTNS*$^{-/-}$) cells after 24 h of incubation in fed and starved condition, respectively (n = 3).

D–J Cell viability test in *CTNS*^{WT}, *CTNS*$^{-/-}$ cells treated with the increasing concentrations of cysteamine, bicalutamide, luteolin, genistein, 8-bromo-cAMP, disulfiram, and a combination of cysteamine and bicalutamide (100 and 35 μM, respectively), respectively (n = 3).

Data information: Data are expressed as mean ± SEM.

 © 2021 The Authors

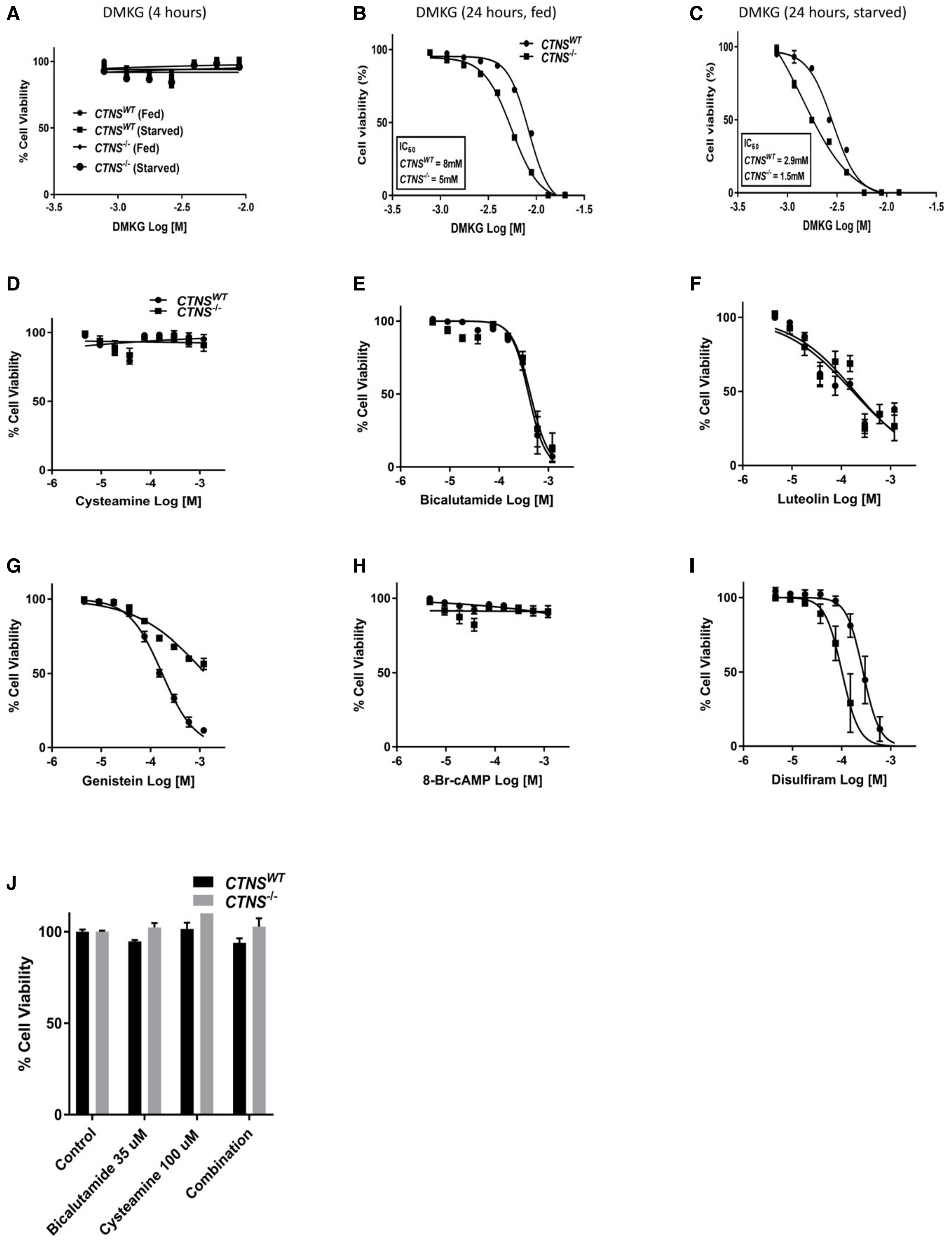
A
DMKG (4 hours)
% Cell Viability
DMKG Log [M]
CTNS^WT (Fed)
CTNS^WT (Starved)
CTNS^-/- (Fed)
CTNS^-/- (Starved)
B
DMKG (24 hours, fed)
Cell viability (%)
DMKG Log [M]
CTNS^WT
CTNS^-/-
IC50
CTNS^WT = 8mM
CTNS^-/- = 5mM
C
DMKG (24 hours, starved)
Cell viability (%)
DMKG Log [M]
IC50
CTNS^WT = 2.9mM
CTNS^-/- = 1.5mM
D
% Cell Viability
Cysteamine Log [M]
E
Bicalutamide Log [M]
F
Luteolin Log [M]
G
Genistein Log [M]
H
8-Br-cAMP Log [M]
I
Disulfiram Log [M]
J
% Cell Viability
Control
Bicalutamide 35 uM
Cysteamine 100 uM
Combination

Figure EV3.

Figure EV4. Cysteamine–bicalutamide combination treatment shows a synergic effect in treatment of cystinotic ciPTEC.

A–C Volcano plot illustrates significantly differentially abundant proteins (n = 3). The -$\log_{10}$ (Benjamini–Hochberg corrected P-value) is plotted against the $\log_2$ (fold change: *CTNS*$^{-/-}$ no drug treatment/*CTNS*$^{-/-}$ cysteamine treatment), (fold change: *CTNS*$^{-/-}$ no drug treatment/*CTNS*$^{-/-}$ bicalutamide treatment) and (fold change: *CTNS*$^{-/-}$ no drug treatment/*CTNS*$^{-/-}$ combination treatment), respectively. The non-axial vertical lines denote ±1.5-fold change while the non-axial horizontal line denotes $P = 0.05$, which is our significance threshold (prior to logarithmic transformation).

D Western blotting and densitometric analyses for LC3-II/LC3-I ratio in CRISPR-generated *CTNS*$^{-/-}$ cells treated with cysteamine (100 μM), bicalutamide (35 μM), and a combination of cysteamine and bicalutamide (100 and 35 μM, respectively). β-Actin was used as a loading control (n = 3).

E Quantification of TFEB-GFP nuclear translocation in *CTNS*^{WT}, and *CTNS*$^{-/-}$ cells upon treatment with bicalutamide (35 μM) (n = 3).

F *TFEB* mRNA expression of the *CTNS*^{WT} cells upon starvation and treatment with bicalutamide (35 μM) (n = 3).

Data information: Data are expressed as mean ± SEM. *P-values < 0.05 were considered to be significant. One-way ANOVA with Dunnett's correction (D, E and F) or unpaired t-test (E). Exact P-values and statistical tests are listed in Appendix Table S1.

© 2021 The Authors

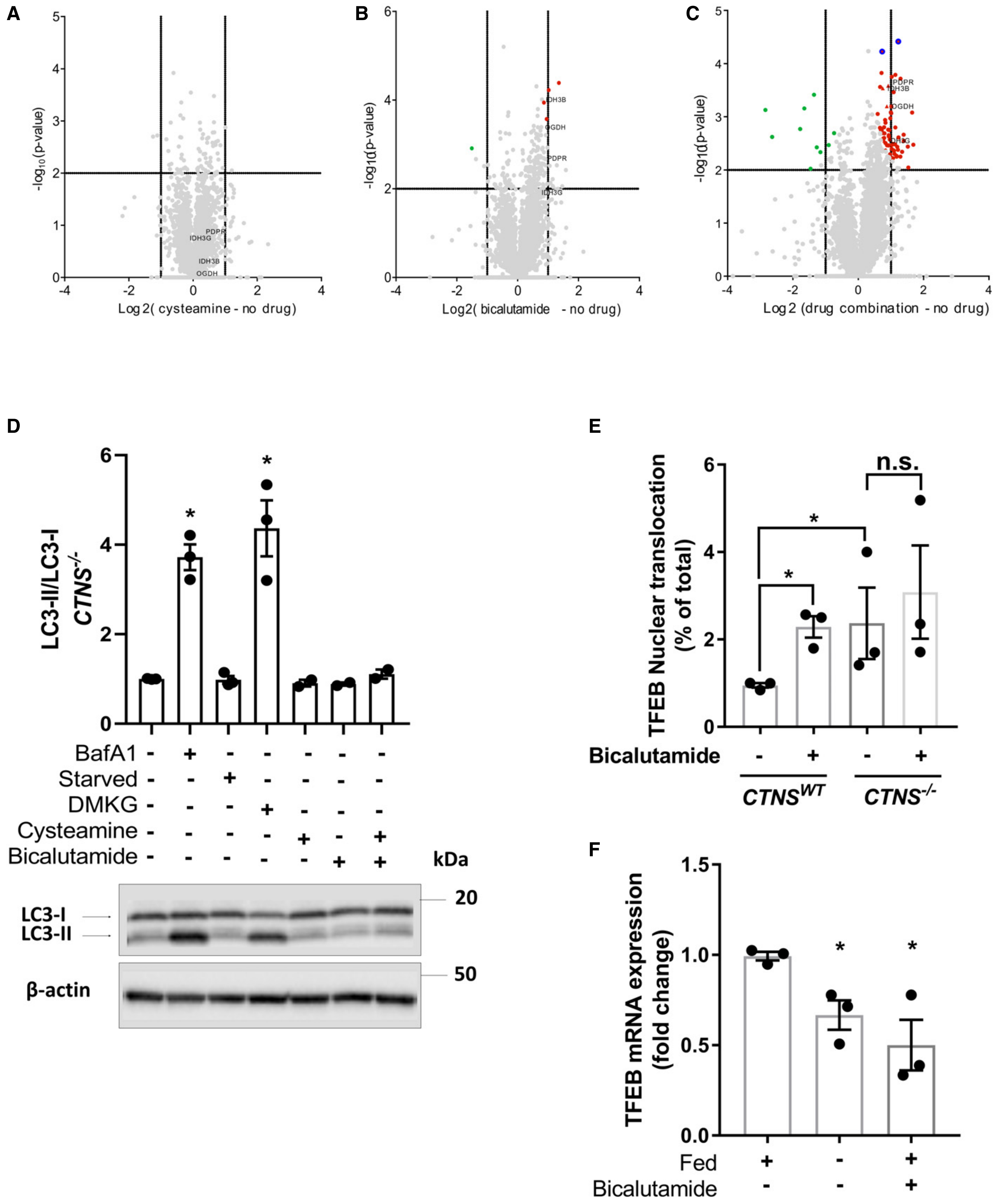
A
B
C
Log 2(cysteamine - no drug)
Log2(bicalutamide - no drug)
Log 2 (drug combination - no drug)
PDPR
IDH3G
IDH3B
OGDH
D
LC3-II/LC3-I
CTNS-/-
BafA1
Starved
DMKG
Cysteamine
Bicalutamide
kDa
20
50
LC3-I
LC3-II
β-actin
E
TFEB Nuclear translocation (% of total)
n.s.
Bicalutamide
CTNSWT
CTNS-/-
F
TFEB mRNA expression (fold change)
Fed
Bicalutamide

Figure EV4.

Figure EV5. Cysteamine-bicalutamide combination treatment is safe in patient-derived cystinotic kidney tubuloids and in wild-type zebrafish.

A Patient-derived cystinotic tubuloids ($CTNS^{Patient\text{-}1}$ and $CTNS^{Patient\text{-}2}$) and tubuloids established from healthy kidney tissue ($CTNS^{WT\text{-}1}$ and $CTNS^{WT\text{-}2}$) were differentiated for 7 days and analysed by quantitative real-time PCR for markers of various segments of the nephron (n = 1).

B Brightfield images of cystinotic tubuloids and healthy control tubuloids at the start of treatment and after 5 days of cysteamine (100 µM)-bicalutamide (35 µM) combination treatment or treatment with medium only (negative control). Scale bars are 2,000 µm.

C, D Bicalutamide safety screening in cystinotic tubuloids. Tubuloid viability upon treatment with cysteamine (100 µM) in combination with increasing concentrations of bicalutamide was compared to treatment with cysteamine alone (= 100% viability) (per donor n = 4 replicates for each dose).

E Survival rates in wild-type zebrafish upon treatment with bicalutamide (10 µM), and a combination of cysteamine and bicalutamide (1,000 and 10 µM, respectively) (n = 40 embryos per group).

F Deformity rates in wild-type zebrafish upon treatment with bicalutamide (10 µM), and a combination of cysteamine and bicalutamide (1,000 and 10 µM, respectively) (n = 40 embryos per group).

G Hatching rates in surviving wild-type zebrafish evaluated at 72- and 96-h post-fertilization (hpf) with bicalutamide (10 µM), and a combination of cysteamine and bicalutamide (1,000 and 10 µM, respectively) (n = 40 embryos per group). Drugs were administered at 48-h post-fertilization in all experiments dissolved in the swimming water with the specified concentrations.

Data information: Data are expressed as mean ± SEM.

 © 2021 The Authors

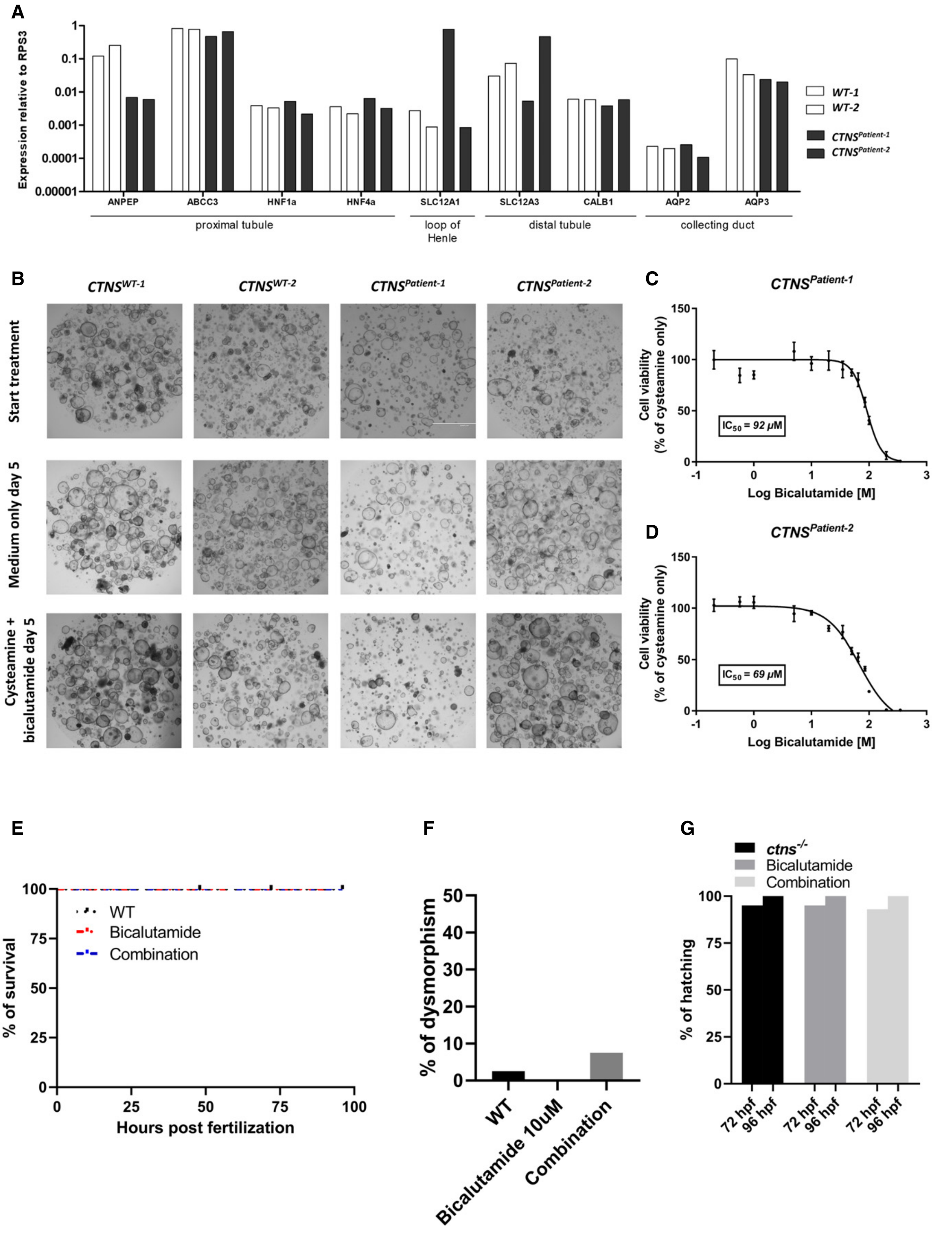
A
Expression relative to RPS3
1
0.1
0.01
0.001
0.0001
0.00001
ANPEP
ABCC3
HNF1a
HNF4a
SLC12A1
SLC12A3
CALB1
AQP2
AQP3
proximal tubule
loop of Henle
distal tubule
collecting duct
WT-1
WT-2
CTNS Patient-1
CTNS Patient-2
B
CTNS WT-1
CTNS WT-2
CTNS Patient-1
CTNS Patient-2
Start treatment
Medium only day 5
Cysteamine + bicalutamide day 5
C
CTNS Patient-1
Cell viability (% of cysteamine only)
IC50 = 92 μM
Log Bicalutamide [M]
D
CTNS Patient-2
IC50 = 69 μM
E
% of survival
Hours post fertilization
WT
Bicalutamide
Combination
F
% of dysmorphism
WT
Bicalutamide 10uM
Combination
G
ctns-/-
Bicalutamide
Combination
% of hatching
72 hpf
96 hpf

Figure EV5.
