# Supplementary material for: Cysteamine–bicalutamide combination therapy corrects proximal tubule phenotype in cystinosis
Source: EMBO Mol Med. 2021 Jun 24;13(7):e13067. doi: 10.15252/emmm.202013067 (PMC8261496; doi:10.15252/emmm.202013067)
Supplement: Supplementary file 6 — Source Data for Figure 1 [file EMMM-13-e13067-s008.pdf]

Figure 1G; for control and CTNS-/- cells

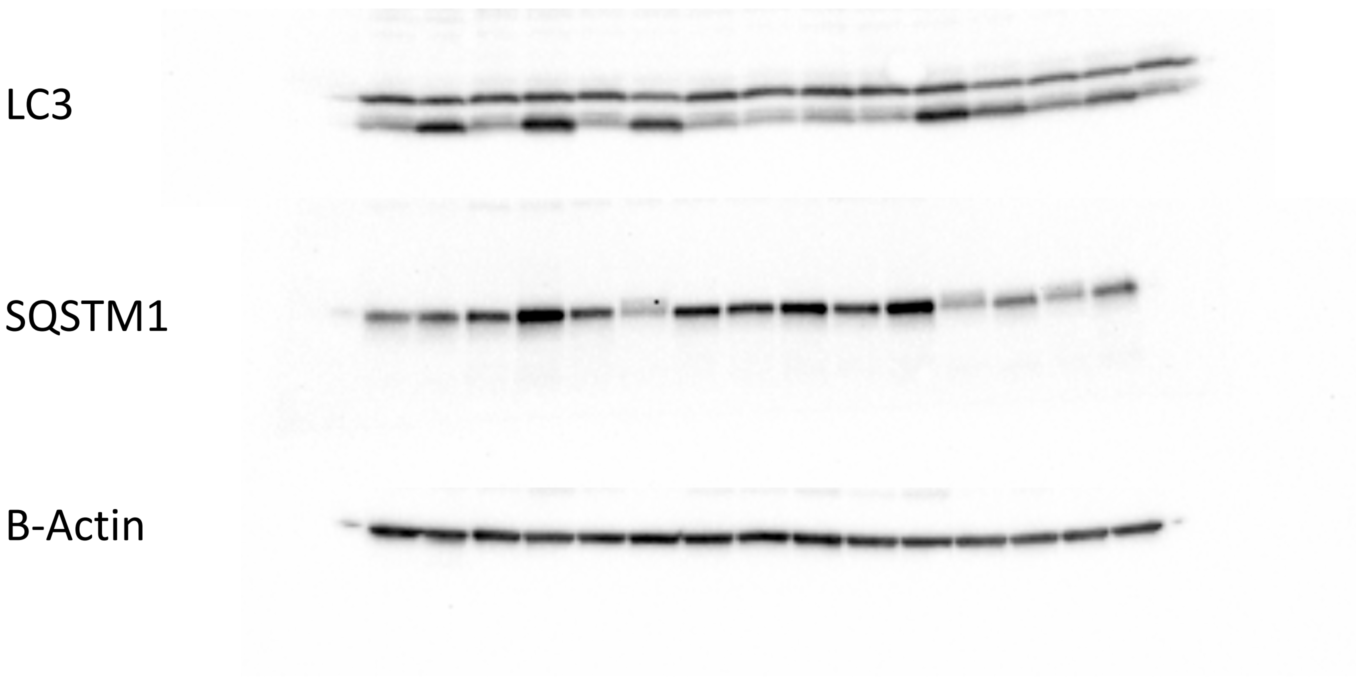

Control (14,4) and CTNS$^{-/-}$ (3) cll lines blot	14,4 CM	14,4 CM + BafA1	3 CM	3 CM + BafA1	3 HBSS	3 AKG	3 BIC	3 CYS	3 BIC + CYS	3 CM	3 CM + BafA1	3 AKG	3 AKG + BIC	3 AKG + CYS	3 AKG + BIC + CYS

CM= Fed condition
HBSS= Starvation
AKG= Alpha ketoglutarate
BIC= Bicalutamide
Cys= Cysteamine

Figure 1G; for control and $CTNS^{Patient}$ cells

LC3

SQSTM1

B-Actin

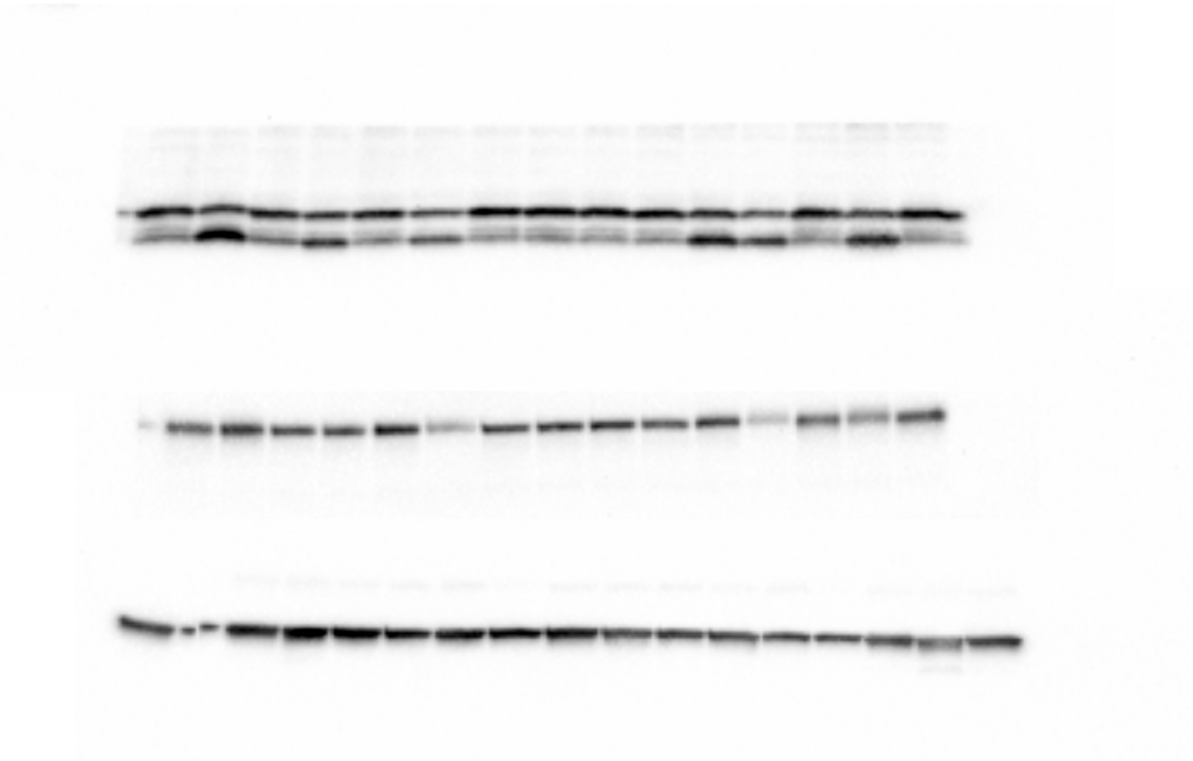

Control (14,4) and $CTNS^{Patient}$ (46,2) cll lines blot	14,4 CM	14,4 CM + BafA1	46,2 CM	46,2 CM + BafA1	46,2 HBSS	46,2 AKG	46,2 BIC	46,2 CYS	46,2 BIC + CYS	46,2 CM	46,2 CM + BafA1	46,2 AKG	46,2 AKG + BIC	46,2 AKG + CYS	46,2 AKG + BIC + CYS

CM= Fed condition
HBSS= Starvation
AKG= Alpha ketoglutarate
BIC= Bicalutamide
Cys= Cysteamine
