# Supplementary material for: Cysteamine–bicalutamide combination therapy corrects proximal tubule phenotype in cystinosis
Source: EMBO Mol Med. 2021 Jun 24;13(7):e13067. doi: 10.15252/emmm.202013067 (PMC8261496; doi:10.15252/emmm.202013067)
Supplement: Supplementary file 8 — Source Data for Figure 5 [file EMMM-13-e13067-s006.pdf]

Figure 5B; for CTNS$^{-/-}$ cells

LC3

B-Actin

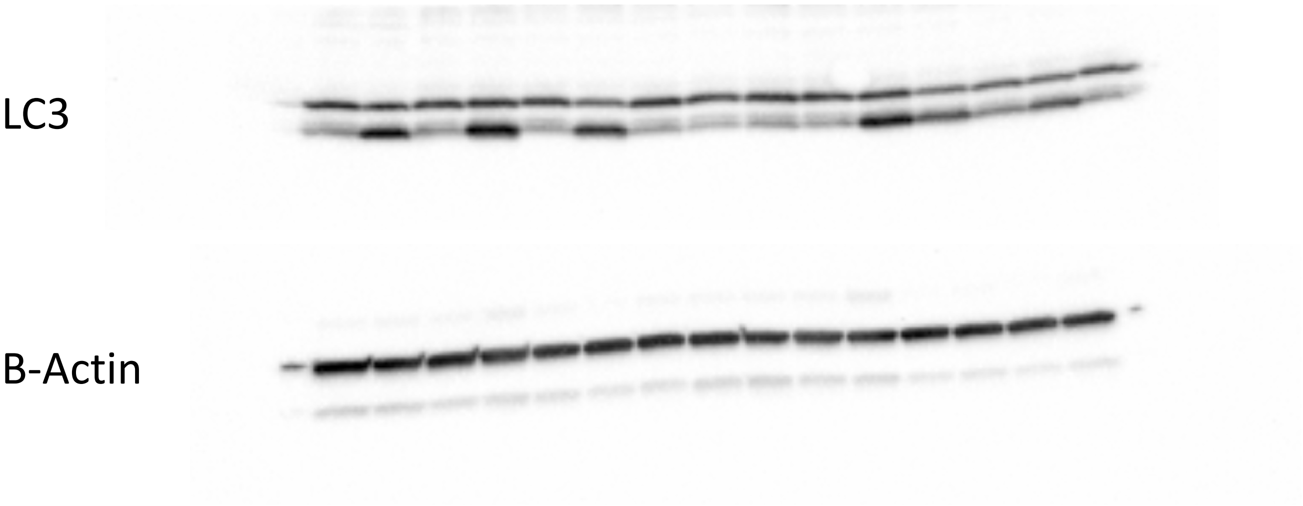

control (14,4) and CTNS-/- (3) cells blot	14,4 CM	14,4 CM + BafA1	3 CM	3 CM + BafA1	3 HBSS	3 AKG	3 BIC	3 CYS	3 BIC + CYS	3 CM	3 CM + BafA1	3 AKG	3 AKG + BIC	3 AKG + CYS	3 AKG + BIC + CYS

CM= Fed condition
HBSS= Starvation
AKG= Alpha ketoglutarate
BIC= Bicalutamide
Cys= Cysteamine
